# Recursive neural networks in hospital bed occupancy forecasting

**DOI:** 10.1186/s12911-019-0776-1

**Published:** 2019-03-07

**Authors:** Ekaterina Kutafina, Istvan Bechtold, Klaus Kabino, Stephan M. Jonas

**Affiliations:** 1 Department of Medical Informatics, Uniklinik RWTH Aachen, Pauwelsstrasse 30, 52057 Aachen, Germany; 2 AGH University of Science and Technology, Faculty of Applied Mathematics, al. Mickiewicza 30, 30-059 Krakow, Poland; 3 St. Marien- und St. Annastiftskrankenhaus, Salzburger Straße 15, 67067 Ludwigshafen, Germany

**Keywords:** Hospital bed occupancy, Time series forecasting, Recurrent neural networks, NARX

## Abstract

**Background:**

Efficient planning of hospital bed usage is a necessary condition to minimize the hospital costs. In the presented work we deal with the problem of occupancy forecasting in the scale of several months, with a focus on personnel’s holiday planning.

**Methods:**

We construct a model based on a set of recursive neural networks, which performs an occupancy prediction using historical admission and release data combined with external factors such as public and school holidays. The model requires no personal information on patients or staff. It is optimized for a 60 days forecast during the summer season (May–September).

**Results:**

An average mean absolute percentage error (MAPE) of 6.24% was computed on 8 validation sets.

**Conclusions:**

The proposed machine learning model has shown to be competitive to standard time-series forecasting models and can be recommended for incorporation in medium-size hospitals automatized scheduling and decision making.

## Background

The main reason for hospital occupancy forecasting is the widely understood need for optimization of resources in a more and more competitive medical field [1, 2]. The better ***predictions*** we can make, the more efficiently we can plan ahead, and as a result, resource use is optimized and, better care can be provided to the patients [3–5].

Hospital bed occupancy forecasting is a topic with multiple scales and perspectives. For example, a problem of optimizing national plans regarding hospital infrastructure means large spatial scale combined with large temporal scale. At a medium scale, hospital staffing and vacations needs to be planned weeks or months in advance to allow a continuous operation with respect to seasonal fluctuations of patients. Alternatively, we can also pose questions about a particular department of a local hospital in an hourly time perspective, for example, to optimize short-term planning in emergency or intensive care units (ICU). Depending on the specific situation and the problem formulation, the choice of methods can vary significantly.

Recent literature has been primarily focused on building mathematical forecasting models for ICUs, as the risks of workflow interruptions are particularly high and the time scale is very small, possibly of the order of minutes [6–9]. On the other hand, the large scale of economic costs encourages hospital occupancy forecasting for medium to long-term planning, that is, in the order of weeks, months or years [10–14].

In this manuscript, we take a look on the medium time scale forecasting of bed occupancy (order of months) for medium scale hospitals (250–500 beds). The data resolution taken into account is 1 day and time periodicity (weekly, seasonally, yearly fluctuations) is assumed to play a key role in the model. Forecasting of occupancy and thereby resource utilization in a medium time scale allows for better allocation of personnel, especially during public or school holidays, or other periods of interest.

There is relatively little information about existing solutions to similarly formulated problems. In Mackay et al. one may find a broad overview of existing models of hospital forecasting from very simple to more modern and complex [15]. Many of these models are using estimates of the length of stay (LOS) [16, 17]. Here however, we are presenting more direct time series - based approach, which requires only historic admission and releases data and no personal patient information.

One of the first applications of time series methods to the hospital bed occupancy problem was a work by Farmer et al. [10], who suggested a stochastic approach and used autoregressive integrated moving average (ARIMA) modelling with Box-Jenkins methodology [18]. This class of models has a long history of business applications, such as stock market simulations [19], and a solid mathematical background [20].

In the recent decades, data-driven approaches and machine learning proved their efficiency for forecasting tasks [21–23]. However, only limited progress was made in applying these methods to hospital bed occupancy forecasting. Length of stay (LOS) [16, 24–27], discharge [28] and readmission [29] forecasting models based on machine learning have been developed. Joy and Jones [30] proposed a combination of ARIMA with artificial neural networks (ANN) to model time series bed occupancy data. However, the ANN part is responsible only for residual estimations.

To our best knowledge, by the time of submitting this paper, no scientific work describing machine learning framework as a main forecasting model for time series data on bed occupancy is currently available.

The primary goal of this paper is to develop a computational model, which uses historic data on daily admissions and releases, combined with external, publicly available supporting data to return a forecast for an upcoming period. We explore the advantages of recurrent neural networks and show that they can be successfully used for medium term hospital bed occupancy forecasting.

## Methods

The aim of this work is the forecasting of hospital bed occupancy. Therefore, a predictive model based on recurrent neural networks has been developed. The focus of optimization was set on the scheduling of medical personnel within seasonal demand fluctuations. In the use case upon which the model is based, a number of clinics share their bed pool in units of 30 beds. Units can be closed or opened based on current occupancy. Each unit requires individual personnel. Therefore, in practice, the ultimate target for forecasting is a correct estimation of the number of units needed at any point in time.

The requirement imposed by hospital management representatives was a sound prediction of bed occupancy for 60 days starting on the first day of the main holiday season months: May, June, July and August.

### Dataset

Our work is based on the hospital records of a medium size German hospital (approx. 400 beds) in the period from October 14th 2002 to December 31st 2015 (4827 days). Each record consists of a patient identifier, time of admission and discharge, and the name of the clinic the patient was admitted to. No personal information on the patients or staff was provided. A total number of 353,520 records were available. A list of clinics sharing their bed pool is provided separately.

### Curation, preprocessing and supporting data

Data preparation is performed in 5 steps: (1) curation by removal of missing values, (2) curation by removal of non-necessary data, (3) data transformation, (4) addition of supporting data, and (5) separation of training, testing and evaluation data.

#### Data curation

In step 1 (removal of missing values), entries with missing data are deleted from the records (i.e., missing date or patient number). These entries correspond to about 2% of the overall data, so the removal will not essentially affect the model. Next, removal of non-necessary data (step 2), the data from clinics not taking part in the bed pool is removed (i.e., ICUs or specialty clinics not in the shared bed pool). These entries correspond to about 50% of the overall data.

#### Data transformation

**Fig. 1:**
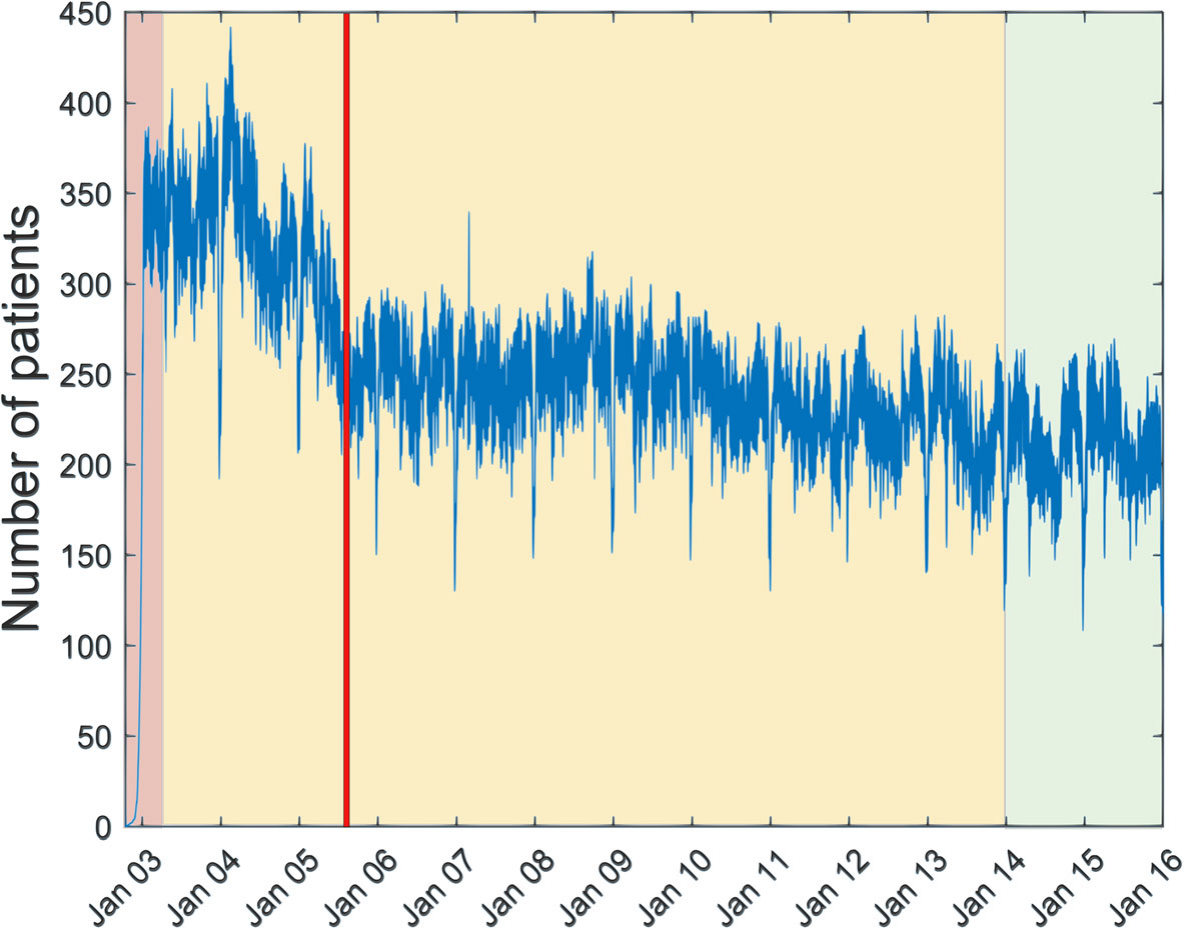
Plot of bed occupancy through the time period covered by the dataset. Only shared beds are shown. The buildup of the occupancy is seen in the first days (pink background) and a stable occupancy is reached by April 2003. Additionally, a restructuring of available beds in the clinics in 2005 can be observed by a drop in occupied beds (red bar/line). Yellow background corresponds to the training and testing data and green background to the evaluation data

Step 3 is needed, since the original data does not directly contain information on the bed occupancy, that is, the number of beds occupied per day. Instead, it contains admission and discharge times for individual patients. In order to construct a time series of daily occupancy, the number of occupied beds in the first day is required. As this number is not available, we assume it to be zero. To compensate for the error, we remove the first 168 entries of the time series data (or, equivalently, remove the data in the period before April 1st 2003, or 168 days). Since we only take full records into account, each patient included in the construction of the time series has an admission and discharge date assigned. Therefore, the patients admitted before 14 October 2002 are not included in the time series but their number becomes neglectable by the chosen date of 1 April 2003 (Fig. 1). The final result of this step is a univariate time series of bed occupancy corresponding to the period 1 April 2003 to 31 December 2015 in the form of a vector with the length of 4657 days.

#### Supporting data

Next, addition of supporting data (step 4) was performed. After an analysis with the hospital managers, the following supporting data vectors were added to the model, as they are likely to influence the bed occupancy:

- **Day of the week.** The day of the week is encoded as numbers from 1 to 7 corresponding to the days from Monday to Sunday. This variable facilitates the modeling of the weekly periodicity.
- **Day of the year.** A day of the year is encoded as numbers from 1 to 365/366 corresponding to the period from January 1st to December 31st. The variable reflects the time of year.
- **Public holidays**. The binary vector indicates whether a given date is a public holiday (binary “yes”) or not (binary “no”). Since the hospital is located on the border of three German federal states and some of the holidays in Germany are state-specific, we added one vector for each state.
- **School holidays.** The school holidays are encoded in the same way as the public holidays with three different vectors for the three states.

The holiday data was extracted from the website of the Standing Conference of the Ministers of Education and Cultural Affairs (Kultusministerkonferenz), the organization publishing the school holidays in Germany [31].

**Fig. 2:**
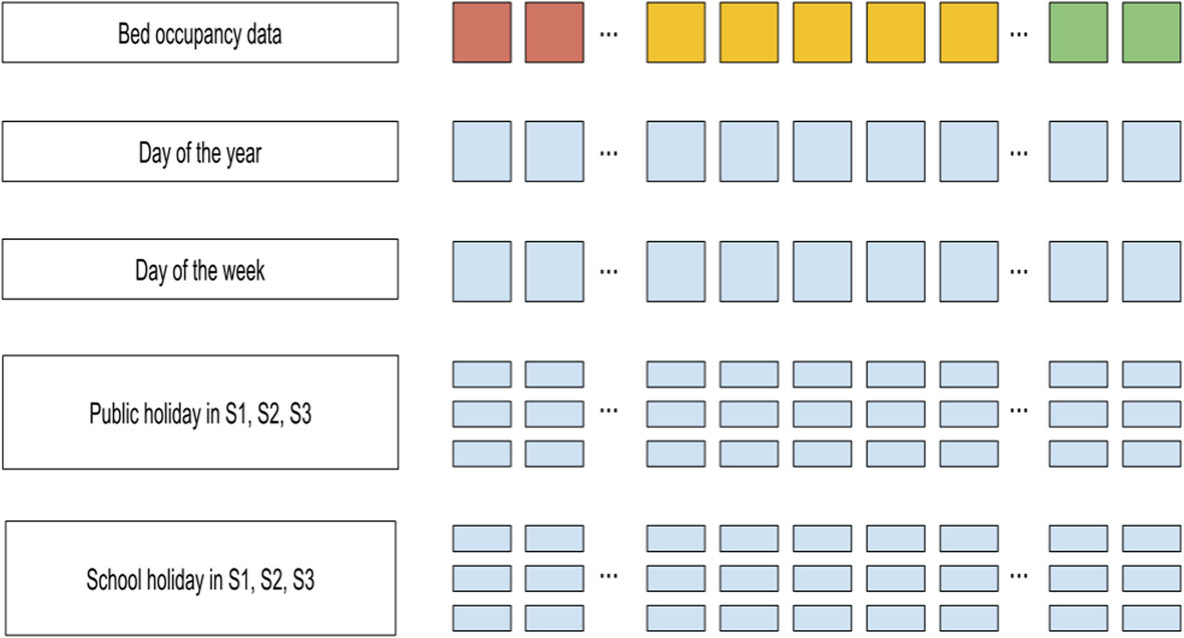
Illustration of the 9-dimensional feature vector. S1, S2, S3 denote three German federal states. Pink, yellow and green colors correspond to the build-up, training and evaluation data. The colors correspond to phases displayed in Fig. 1

The supporting data (day of the week, day of the year, national and school holidays, prospective and retrospective information) is formatted to form multivariate time series, aligned with the bed occupancy univariate time series. It is arranged in a matrix with 9 rows and 4657 columns, corresponding to the days in the period from April 1st 2003 to December 31st 2015 (Fig. 2).

The final data set to build the model consists of bed occupancy and supporting data describing external conditions. Please note that the supporting data can be assessed for future time intervals during forecasting, while the bed occupancy data can be only known for the past.

#### Separation of training, testing and evaluation data

The cleaned data is divided into two parts. The first part (training and testing data) covers the period from April 1st 2003 until December 31st 2013. This part is used for tuning the model parameters. The second part (1 January 2014 to 31 December 2015) is used exclusively for model validation (evaluation data) and is used only once for validation of the prediction quality on unseen/new data.

The training and testing data are split into separate folds for training and testing sets. Each of the months May to August for the years 2009 until 2013 are used for a test-prediction during parameter optimization (20 test cases total) and for each test case, the period of 1 to 5 years prior to this time point is used as training data.

### NARX model

While still rarely referred to in the healthcare management forecasting context, machine learning methods such as Artificial Neural Networks, Decision Trees, or Support Vector Machines were successfully used in engineering and business applications (see [21] for an overview).

Moreover, multiple publications provide comparisons between stochastic models (autoregressive-moving-average (ARMA), autoregressive integrated moving average (ARIMA), seasonal ARIMA (SARIMA), generalized autoregressive conditional heteroscedasticity (GARCH) and a special type of a recurrent Artificial Neural Network: nonlinear autoregressive model with exogenous terms (NARX) [32].

While it is difficult to make generalizations, comparisons based on specific data, such as chaotic laser time series [33], wind speed [34] or refrigeration compressors production [35] tend to agree that NARX are superior to stochastic methods, particularly for multi-step forecasting [33].

The NARX model assumes that the value of the prediction variable (bed occupancy in our case, denoted by *y*) at time *t* is dependent on the local historical values of this variable (last *d*_*y*_ measurements) as well as on the local values of the external variable (in our case, 8-dimensional variable of supporting values, denoted by *u* on a time interval covering the last *d*_*u*_ measurements:

1 $$ y(t)=f\left(y\left(t-1\right),y\left(t-2\right),\dots, y\left(t-{d}_y\right),u\left(t-1\right),u\left(t-2\right),\dots, u\left(t-{d}_u\right)\right). $$

The length of the “memory” parameters *d*_*y*_ and *d*_*u*_ influences the complexity of the model and the computing time. For simplification we assume, that *d*_*y*_ *= d*_*u*_ *= d.* We will further refer to the parameter *d* as “delay”. The value of this local history parameter is chosen during parameter optimization.

Equation (1) describes the essence of the model, which we propose here. Apart from the parameter *delay d* described above, a number of parameters are needed to specify the function *f*. Here, function *f* represents a neural network with *hL* hidden layers and a fixed number of *hN* nodes per layer (for simplification). During forecasting, the described neural network becomes a recurrent system, since the output data for *y(t)* is used as an input for the next steps, thus the name recurrent neural network.

### NARX training and testing

In order to structure the modelling process, we define a four-step training and testing procedure, which is subsequently used to iteratively optimize the model’s parameters and to make a forecast about the future hospital bed occupancy. The overall evaluation of the performance is done afterwards using the evaluation data.

**Fig. 3:**
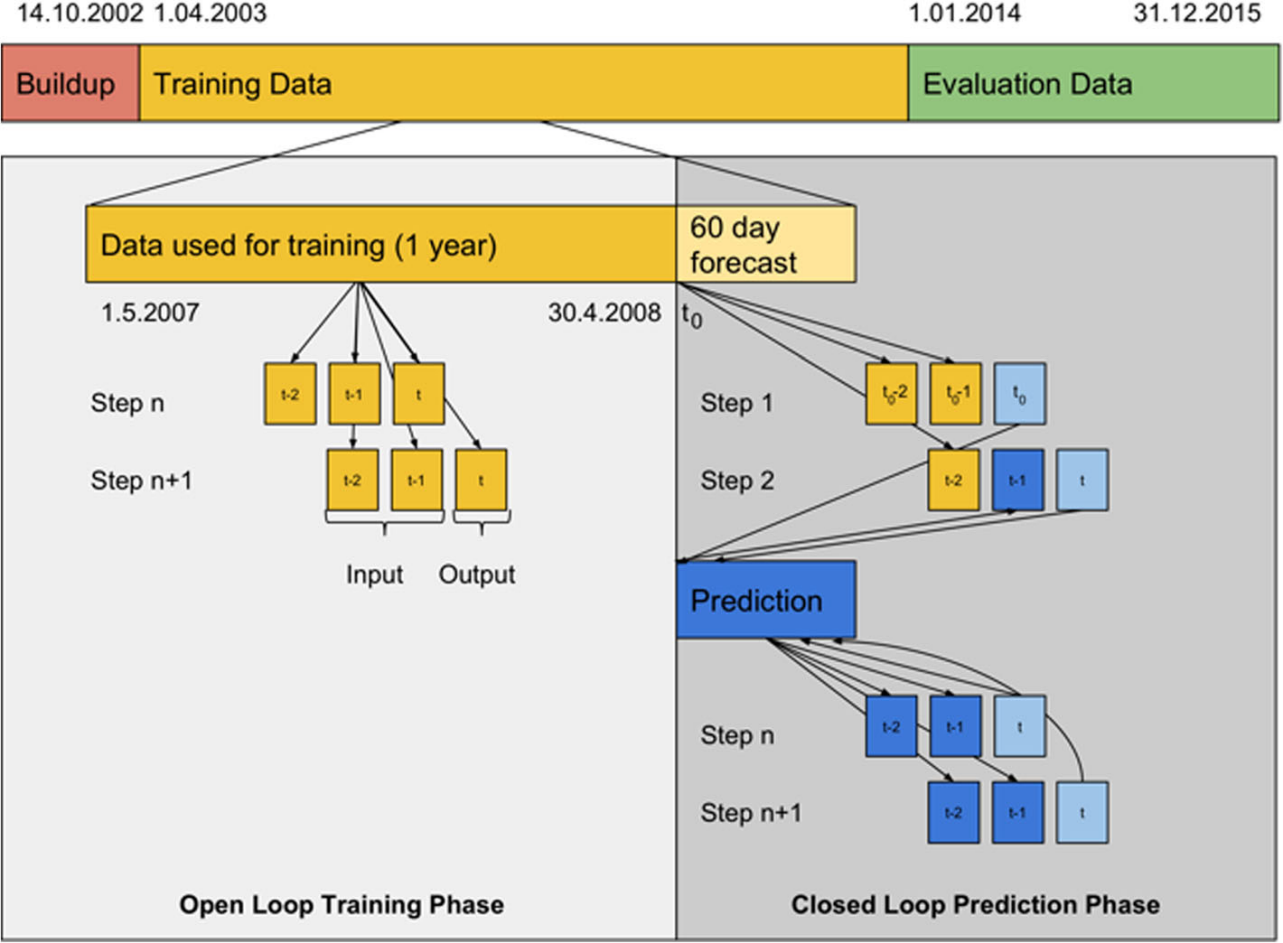
Open loop training and closed loop prediction of NARX on an example sequence of bed occupation data with delay d = 2. Prediction and training is handled identically for training and evaluation data. Supporting data is not shown for simplicity, as it is always taken from pre-calculated time series (top bar) and never from prediction

The NARX model uses a so-called open loop during training and a closed loop during testing. This means that during training all information is taken from the feature vector, while during testing, part of the data is fed back from the network to itself (Fig. 3).

As an example, if a history of 1 year and a delay of 2 is used to predict 60 days starting May 1st 2008, the following data would be used. Training data would be the data of the 365 days prior to May 1st 2008, which is May 2nd 2007 (due to leap year) to April 30th 2008. For the prediction of the first day (May 1st 2008), the bed occupancy data of April 29 and April 30 2008 are used as input for the NARX. For the second day (May 2nd 2008), the first prediction (May 1st 2008) and the occupancy at the last day of April is used as input. In the next step, only the newly predicted occupancies are used (closed loop).

### Step 1: Fix model parameters

Choose the first day of the 60-days long period for a forecast.

In the time series data, it corresponds to a certain natural number *t*_0_ and *t*_0_*-d* must belong to the period included in the dataset. The following parameters need to be fixed for the following training process:

- delay *d = d*_y_ = *d*_u_ (see eq. (1)): delay of the model. It describes how many days in the past are assumed to influence the day *t*_0_.
- history *k*: length of history chosen for the training procedure. In other words, we take a *k*-year subset of the data into account (e.g., the last year), instead of all previous data.
- *hL* - number of hidden layers.
- *hN* - number of nodes in each hidden layer.

### Step 2. Training of the model

To train the NARX model, the data for the individual training cycle needs to be selected (see Fig. 3). The set of data points is prepared as follows: to each date *t*_i_ ∈ *[t*_0_*-k*, *t*_0_*–1]* corresponds the input vector *(y(t*_i_*-d)*,..,*y(t*_i_*-1)*, *u(t*_i_*-d)*,...,*u(t*_i_*))* and the output y(*t*_i_). Please note that since *t*_0_*–1* must belong to the dataset and is the last point of the training data, the values *y* are known for each item in the training data. The neural network is trained using the backpropagation algorithm.

### Step 3. Forecast/testing of the model

The previously trained NARX model is used to forecast a 60 days long time period *(t*_0_, *t*_0_ + *60)* (see Fig. 3). This part must be performed recurrently, since, independently of the choice of *t*_*0*_, we assume that the future values *(y(t*_0_),...,*y(t*_0_ + *60))* are unknown. On the other hand, the supporting data *u* is available at any time step, including future time steps. In the first step of the forecasting we take the input vector *(y(t*_0_*-d)*,..,*y(t*_0_*–1)*, *u(t*_0_*-d)*,...,*u(t*_0_*))* and receive an output $\widehat{y}$(*t*_0_), which is used to form the next input vector. After repeating the procedure *60* times, we obtain the *60-*step forecast *(*$\widehat{y}$*(t*_0_*)*,..., $\widehat{y}$*(t*_0_ + *60–1))*.

### Step 4. Ensure robustness

The backpropagation algorithm for network training used in our work is based on the initial randomization of the NARX model parameters and iteratively optimized during training. Therefore, the predictions can potentially vary substantially. In order to prevent this phenomenon, the randomization seed could be fixed, which would make the results fully reproducible. On the other hand, particular seeds can also produce a very large error.

In order to stabilize the prediction without losing the advantage of the randomization we decided to repeat the steps 2 and 3 multiple times (here 50) and average the results. This linearly increases the computation time, but makes the results more reliable.

### Errors

There are various types of ways to evaluate the quality of the prediction. Typically, an error of the forecast is defined as a certain statistic of error vector *e*_*i*_ *= |y(t*_i_*)-*$\widehat{y}$*(t*_i_*)|, t*_i_*∈ [t*_0_*,t*_0_ *+ 60–1],* where *y* denotes an actual value of the bed occupancy variable and $\widehat{y}$ - the predicted value.

In this work, the following metrics were chosen:

1. **MAX** = *max*(| *e*_i_| , *i* = 1..60). Maximum error carries the information about the worst prediction within the 60 days forecasting period.
2. **MAE** = *mean*(| *e*_i_| , *i* = 1..60). Mean absolute error gives an overall picture about the quality of the prediction.
3. **MAPE**=100 ∗ *mean*(| *e*_i_/*y*_i_| , *i* = 1..60). Mean absolute percentage error value shows the averaged error expressed as percentage. Often used in similar problems [11] and thus facilitating the comparison.
4. **RMSE** = $\sqrt{1/n{\sum}_{i=1}^{60}{e_i}^2}$ . Root-mean-square error. Similarly to MAPE, facilitating the comparison.
5. **GE** =$\mathit{\max}\left( floor\left(\widehat{y}/30+1\right)- floor\left(y/30+1\right)\right),i=\mathrm{1..60}\Big)$. This is a problem-specific error. As it was mentioned before, hospital clinics are sharing beds in groups of 30. It is important that during the forecasted period, the number of actually needed groups is as close as possible to the predicted number. Moreover, large differences on just 1 day can be considered as a failure of the forecast even if the prediction is good on average. GE can take natural values, one and zero. This captures the maximum difference between actually needed and predicted units, both too many and too few. Any positive numbers indicates that either one or more units were empty or too few beds were available on at least 1 day during the forecasting period.

Further on we prioritize MAE error for the optimization procedures. The reason is that MAE reflects the overall picture (unlike MAX, which can be drastically influenced by 1 day fluctuations) and is less prone to small changes than GE (problem of the threshold-based functions where one bed can make a large difference).

In comparison to MAPE and RMSE, MAE has a more intuitive interpretation, while similar in most other characteristics. Later, we will see that despite minor differences, all four types of errors show a consistent picture.

### Parameter optimization

In the current model we have the following four degrees of freedom: k, hL, hN, d (see Step 1 and 4 above for the description). While establishing the global optimum in the full parametric space would have very high computational costs, local optima could be found reasonably fast. In order to do so we will make use of the following optimization procedure.

1. **Optimize training data length k and number of hidden nodes hN.**
  - We assume the following values of other parameters: hL = 1and d = 2,. For every pair of parameters hN∈{1,5,10,15,...,40} and k∈ {365,730,1095,1460,1825} (1–5 years) the basic forecasting procedure is applied to the dates 1 {May, June, July, August} 2009–2013.
  - Five described above types of errors are computed for a given date, value of k and hN.
  - For fixed pair (k, hN) the errors are averaged along the dates.
  - Optimal pair (k_0, hN_0) is chosen based on the minimal value of mean average error (MAE).
2. **Optimize number of hidden layers hL.**
  For (k_0, hN_0) we similarly check the results for different numbers of layers (hL) and fix the optimal value hL_0.
3. **Optimize delay d, the local history used for prediction.**
  For (k_0, hN_0, hL_0) optimization of the last parameter d is made.

This process is repeated and the prior resulting configuration of the parameters is used as initial parameters for the next iteration until the parameters stabilize and do not change within one iteration anymore. As a result, a local optimum of the parameter settings has been found.

### Model evaluation

For the previously chosen optimal parameter set, the basic forecasting procedure is applied to the summer seasons of the years 2014–2015. We perform 60-days long prediction for 1st of May, June, July, and August, and compute the five types of errors. Finally, the full yearly prediction starting on 1 January will be performed for the years 2009–2015 to show the model transferability to the different conditions, such as rapid changes related to the major holidays.

### Software

MATLAB R2017a (The Mathworks, Natick, MA, USA) was used to perform the computations. The NARX was implemented using the Neural Network Toolbox.

## Results

### Optimization of the parameters

Best results for a 60 days long prediction period starting on the first days of the summer season months May to August 2009–2013 could be achieved using a training data length of 1 year (k = 365), a delay of 2 days (d = 2) and two hidden layers with two nodes each (Table 1). An MAE of 12.1 beds was calculated. No significant differences were observed between MAE on individual days of the week. Notably, longer history of training data could not achieve a better result (Table 1). A prediction period of 1 year (365 days) resulted in an average MAE of 15.65 (±2.65) (Table 2). When applying the optimal parameters to the summer season months of the evaluation data, an average MAE of 12.51 (±2.54) was achieved (Table 3).

**Table 1:** Overview of training results with different history lengths

History length	MAPE (%)	MAE	MAX	GE	RMSE
1 year	**5.48**	**12.10**	**36.72**	**1.35**	**14.99**
2 years	5.80	12.66	37.74	1.40	15.67
3 years	5.87	12.81	37.66	1.40	15.77
4 years	5.80	12.73	38.23	**1.35**	15.75
5 years	6.18	13.52	39.18	1.50	16,59

**Table 2:** Yearly prediction 2009–2015 (starting January 1st, 365 days prediction)

Year	MAPE (%)	MAE	MAX	GE	RMSE
2009	6.03	14.29	82.45	3	18.65
2010	5.76	12.83	62.79	2	17.13
2011	7.68	16.25	57.04	2	20.58
2012	6.71	14.93	61.20	2	18.95
2013	9.22	20.43	95.30	4	25.17
2014	8.75	17.50	68.12	2	21.51
2015	6.42	13.34	66.56	3	16.73
*Average*	7.22	15.65	70.50	3	19.82
*Standard deviation*	1.35	2.65	13.78	1	2.92

**Table 3:** Results for summer seasons 2014–2015 with determined parameters

Date	MAPE (%)	MAE	MAX	GE	RMSE
*1 May 2014*	*7.08*	*13.80*	*32.99*	*2*	*16.52*
*1 June 2014*	*6.11*	*12.16*	*34.24*	*1*	*14.99*
*1 July 2014*	*4.64*	*8.83*	*29.43*	*1*	*10.84*
*1 August 2014*	5.54	11.10	34.31	1	13.71
*1 May 2015*	*6.27*	*13.87*	*33.19*	*1*	*16.82*
*1 June 2015*	*8.49*	*17.40*	*36.70*	*1*	*19.67*
*1 July 2015*	*5.87*	*11.34*	*30.41*	*1*	*13.53*
*1 August 2015*	5.92	11.58	28.48	1	13.37
*Mean*	6.24	12.51	32.47	1	14.93
*Standard deviation*	1.14	2.54	2.79	0	2.71

**Fig. 4:**
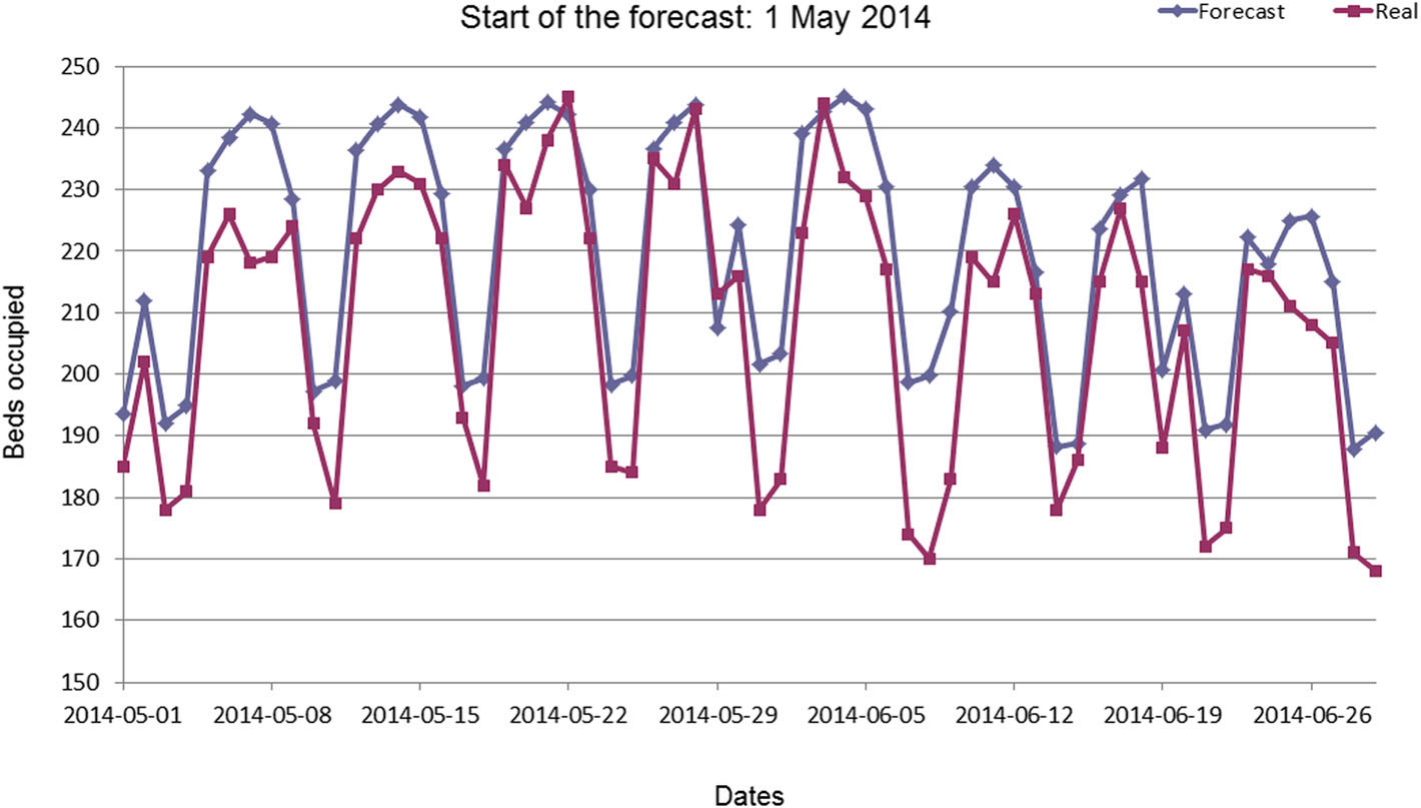
Example prediction and real bed occupation for a 60-day prediction starting in May 2014

**Fig. 5:**
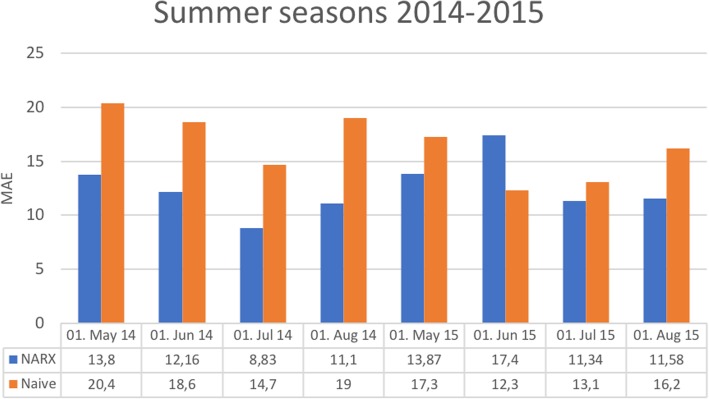
Comparison of MAE for NARX model to naive model for summer seasons 2014–2015

Since the results showed, that only 1 year of history performs best, a natural question is raised: what if we just forecast the same number of beds, which was needed last year on the “same” day? Therefore, a naive approach was implemented that predicts the same amount of beds as 1 year ago. Since the weekday is an important indicator of occupancy (Fig. 4), the bed occupancy from 364 days ago was used instead of 365 days. Thus, the day of the week remains the same. An average MAE of 16.4 (±2.9) was achieved by the naive model, outperforming the NARX only in one of eight predictions (Fig. 5).

To compare our work with the work of Kim et al. [11], the MAPE for a 1 and 30 day prediction period were calculated. For the one-day prediction, all days in the summer period of May to August 2014–2015 were individually predicted. For the 30-day prediction, the first 30 days of each of the summer months May to August 2014–2015 were predicted. The MAPE was 4.01% (±3.16) and 5.55% (±1.21) for 1 and 30 day prediction period respectively.

## Discussion

It is common to predict bed occupancy with respect to the length of stay (LOS) of a single patient. This is based on various information such as age, gender, medical history or medical tests results [26]. In contrast, hospital bed occupation prediction based on time series as performed in this work does not require any personal information of individual patients, hospital staffing or performed procedures. Thus, it can be considered privacy preserving. Yet, a direct comparison between our approach and prior work is not easily possibly. Additionally, no evaluation database is publicly available; therefore the following comparisons can only be an indication of performance. For a definitive evaluation, all methods should be tested on the same dataset.

Kim et al. [11] compare historical averaging to several time series methods (ARIMA, SARIMA and GARCH). The work reports 6 and 8.8% MAPE on correspondingly 1 day and 30 days forecasts. The average MAPE on our testing set is 4.01, 5.55 and 5.48% on 1 day, 30 days and 60 days forecast respectively. This suggests that NARX-based model outperforms the traditional time series approach. Jones et al. [36] report approximately 15 beds RMSE over a prediction period of 32 days, with the average of total bed occupancy around 440 beds. Our approach achieved an RMSE of 14.99 and 13.51 on approx. 220 beds in a 60 and 30 day prediction period respectively. In comparison to the previous methods, our solution is in line with the current state of the art or outperforming it on our specific dataset.

One of the most important advantages of the model is its robustness on several levels. First, the model is trained, optimized and validated based upon a relatively large dataset. Second, the search of the optimum is done in systematic way and parameters in proximity to the found optimum appear to have a relatively small gradient. Another indicator for stability is the difference between the errors on the training and validation sets. Here, the difference is small (5.48% vs 6.24% MAPE and 12.10 vs 12.51 MAE on training and validation respectively), which indicates that the model was not overfitting to the training data. One reason is the computation and averaging of multiple models (*N* = 50) at each step and the small model size.

Introducing multiple errors ensures better control on the optimization. The mean absolute error (MAE) was chosen for the optimization purposes, as it reflects the model quality over the whole forecasted period. However, MAPE, MAX and RMSE are following the MAE in most cases. The last type of the error, GE, is less intuitive as it does not directly depend on the daily differences between the prediction and the forecast. The difference can be just one bed, and yet the number of bed groups needed be different. Nevertheless, we report this error because of its importance to the hospital planning.

In general, the results of the evaluation are acceptable and motivate to use the proposed model as a part of the hospital planning system.

Finally, despite the fact that the optimization was performed for 60 days of the summer season, the model works well for whole year forecasting as well.

Perhaps the most surprising result of this paper is that the proposed model predicts optimally using only the information from the previous year, while a much longer history is available. While this has a positive influence on the speed of the computation, other advantages or disadvantages are possible and should be investigated in future. First, due to the small history the model can adapt more quickly to changes in hospital infrastructure (e.g., closing or opening of new wards).

On the other side, some limitations arise from the short history of the proposed model. Local one-time events such as disasters, diseases outbreaks or events with slower periodicity (Olympic Games, soccer championships) might not be predictable and could have a higher influence on the following year. Similarly, Jones [37] reported on/off switching (rapid increase and decrease) of hospital bed occupancy with a cycle length of 2 years, which can also not be modeled with a history of less than one cycle length. The usage of multiple years can have a smoothening effect and reduce the possible error in these cases. However, neither on/off switching nor other events with such an effect could be observed in this work.

There are several possibilities to further improve the model. The most obvious way is to work on the optimization procedure. There are parameters left in the model, which can still be tuned. For example, we assumed that the delays on the internal and external data are equal, but this assumption can be easily removed to get one more degree of freedom.

Another possible factor to take into account is weather, specifically excessive heat or cold, or other external factors such as flu outbreaks. Because of the length of our goal forecast (60 days), which is much longer than reliably available detailed weather forecasts, we decided to not incorporate it in the model. However, with the help of Bayesian modelling [38], this kind of uncertainty can be taken into account.

In case of longer times of prediction, other factors might play role. For instance, Jones [39] suggested number of hospital deaths as a possible bed occupation predictor.

Lastly, prediction intervals could also be a useful addition, as they could allow the user to have information on the uncertainty of the model.

## Conclusions

We presented a mathematical model based on recurrent artificial neural networks, designed to forecast the bed occupancy in hospitals. Recurrent NARX networks were successfully used for time series data modelling in other areas, but to our best knowledge the presented work is the first application of NARX to hospital bed planning. With 6.24 MAPE on 60 days forecast, our model is competitive to the current state of art, while not using any sort of personal patient’s data. Instead, it is based on admissions and releases data only. The model was optimized for the summer seasons and the data from a medium-size German hospital for optimized scheduling purposes. The model is flexible and can be easily adapted to different requirements. In particular, it can be integrated into an automatic decision model, e.g. similar to the one developed by Grübler et al. [40]. Currently, a software with a user-friendly interface and better performance characteristics is being developed as a web-based application for open access.

## Abbreviations

ANN: artificial neural network
ARIMA: autoregressive integrated moving average
ARMA: autoregressive moving average
GARCH: generalized autoregressive conditional heteroskedasticity
ICU: intensive care unit
LOS: length of stay
MAE: mean absolute error
MAPE: mean absolute percentage error
MAX: maximal absolute error
NARX: nonlinear autoregressive model with exogenous terms
RMSE: root-mean-square error
SARIMA: seasonal ARIMA
